# Safe limits on work hours for the nursing profession: a rapid evidence review

**DOI:** 10.3389/fgwh.2024.1455422

**Published:** 2024-10-31

**Authors:** Heather Katherine Scott-Marshall

**Affiliations:** ^1^ Dalla Lana School of Public Health, University of Toronto, Toronto, ON, Canada; ^2^ Social and Behavioural Health Sciences Division, Dalla Lana School of Public Health, University of Toronto, Toronto, ON, Canada

**Keywords:** nursing, health care, long work hours, shift work, occupational fatigue, occupational health risk factors, safety-sensitive work

## Abstract

Persistent staffing shortages in health care driven by years of inadequate funding and deficiencies in human resources planning, which overlooked the impacts of population aging, have converged into a crisis in health care settings. An essential consequence of the widespread and growing staffing shortfalls in health care has been increased pressure on nurses to work longer hours. The present rapid review has two major objectives: (1) to systematically review and synthesize evidence considering the health and human consequences of excessive work hours, work-related fatigue and associated occupational health and safety hazards; and, (2) to identify policies and practices that demonstrate efficacy in managing or mitigating the adverse effects of occupational fatigue. Findings show that shifts lasting longer than 12 h elevate the risk of occupational fatigue, leading to several fatigue-based hazards. Despite governmental restrictions on long work hours and occupational fatigue in safety-critical industries such as transport, aviation, and nuclear sectors, health care remains largely unregulated in this regard. Ensuring safe and high-quality care over the long term requires implementing adequate regulatory supports for work hour limits for nurses. These measures not only improve workplace satisfaction but also enhance patient outcomes, ultimately fostering a healthier and more resilient health care system.

## 1 Introduction

Nurses in Canada are increasingly grappling with significant and mounting challenges in delivering high-quality patient care. Years of inadequate health care funding, compounded by numerous failures in human resources planning that overlooked the impacts of population aging, have coalesced into a crisis in health care settings and nursing working conditions. A persistent driver of this crisis has been chronic staffing shortages, resulting in extended work hours, unsustainable increases in workload, toxic work environments, and clinical burnout (1). The onset of the COVID-19 pandemic exacerbated these pre-existing challenges in health care settings. Since 2020, the first year of the pandemic, there has been a notable rise in the proportion of nurses choosing to change jobs or leave the profession altogether. Many of those who have switched jobs opted to work for private agencies, which raises serious concerns about the long-term viability of the public health care system (2).

Nursing remains a predominantly female profession; at present, approximately nine in ten regulated nurses in Canada are women. A survey undertaken by the Canadian Federation of Nurses Unions in February 2022 found that more than half (53%) of nurses were considering leaving their current position within the coming year. Reasons cited for leaving included burnout, stress, and poor working conditions due to insufficient staffing levels (3). The shortage of nurses has become a major concern for health care systems across Canada, with many hospitals and long-term care facilities struggling to maintain adequate staffing levels. Data from Statistics Canada show that, between 2018 and 2023, job vacancies in nursing increased nearly three-fold from 10,910 to 30,790, indicating that staffing deficiencies will be very difficult to remedy in the near-term (4).

An essential consequence of the widespread and growing staffing shortages in health care is the increased pressure on nurses to work longer hours. Whether explicit through mandatory overtime or implicit through expectations from health care authorities and employers, there is a pervasive belief that nurses should fill staffing gaps by working additional shifts and extended hours. Scheduled shifts can extend up to 16 h and often deviate from the traditional day, evening, and night shift patterns. For instance, while typical 12 -hour shifts might traditionally run from 7:00 a.m. to 7:00 p.m., nurses may now be required to work shifts starting at 3:00 p.m. and ending at 3:00 a.m., depending on specific staffing needs within their unit (5). This variability can significantly impact nurses’ work-life balance and contribute to fatigue and burnout, highlighting the urgent need for effective staffing solutions and supportive work environments in health care settings (5). Nurses working in specialized units such as surgery, dialysis, or intensive care often face the expectation of being available for extra shifts beyond their regular schedules. In settings like emergency rooms, where staffing shortages are particularly severe, 24 h shifts are increasingly common (6). These scheduling practices persist despite evidence indicating that prolonged wakeful periods of 24 h or more can significantly impair cognitive and physical response times, akin to exceeding the legal blood alcohol limit for operating a motor vehicle (7). Extended hours and overwork in nursing, driven by inadequate staffing, are linked to a higher incidence of medical errors and elevated rates of patient morbidity and mortality (7–9). Excessive work hours and fatigue also heighten the risk of workplace accidents and injuries among nurses (10, 11). Furthermore, research also indicates that nurses are at increased risk of motor vehicle accidents due to drowsy driving after their shifts (12).

Despite the increasing evidence highlighting the risks of excessive work hours and occupational fatigue, the health care industry continues to rely on demanding longer hours from a shrinking pool of nurses. The lack of regulation of work hours in nursing starkly contrasts with other safety-sensitive industries like trucking, rail and nuclear where legislative standards strictly enforce work hour limits. This discrepancy raises concerns about an underlying gender-bias in the regulatory regime that must be addressed.

The present rapid review has two objectives: (1) to compile and synthesize evidence examining the health and human consequences of excessive work hours, work-related fatigue and associated occupational health and safety hazards, for the purpose of identifying safety limits for work hours; and, (2) to identify policies and practices that demonstrate efficacy in managing or mitigating the adverse effects of occupational fatigue. Rapid reviews offer a streamlined approach to synthesizing research evidence in a timely manner to deliver concise and relevant information to key decision-makers. The current review was undertaken on behalf of the Canadian Federation of Nurses Unions (CFNU) in an urgent response to the retention crisis among front-line nurses within the public health care sector.

The review proceeds in two main sections. The first is an overview of evidence on the individual and work-related impacts of excessive work hours, work-related fatigue and fatigue-based impairment, aiming to inform safety limits on work hours in nursing and the health care sector. The second section provides a summary of polices, practices and regulations related to work hours safety within safety-sensitive industries outside of health care within Canada and select peer countries (US, EU) to identify best practices.

## 2 Research evidence on the health and safety outcomes of long work hours and occupational fatigue

### 2.1 Method

A rapid review of the peer-reviewed literature on work hours safety was conducted across multiple scientific databases, including: CINAHL, Embase, ProQuest Health, Safety Science Abstracts, Medline, PubMed, Scopus, Web of Science, JSTOR, ScienceDirect, ProQuest, Directory of Open Access Journals, PLOS, and Google Scholar. Because a majority of the research on occupational fatigue safety limits in work hours has been undertaken outside of nursing and health care, topical studies across all industries and work contexts were considered. The search was structured based on the target population (workers in safety-sensitive industries and/or industries operating on a 24 h schedule), the determinant (long or excessive work hours), and the outcome (health and safety consequences of occupational fatigue). Focal keywords for the target population included: safety-sensitive industries [/transportation/trucking/rail/aviation/nuclear/health care], 24/7 operations [/manufacturing]. Key search terms for the determinant included: long [extended/excessive] work hours [/schedule], shift work, [occupational/work-related] fatigue, sleep [deprivation/deficit]. Search terms for outcomes included: occupational health [/and safety], worker health, work culture, organizational outcomes [/effects]. The search period covered approximately three decades, beginning in 1990, during which there was a significant increase in research activity on occupational fatigue, including several seminal studies on the topic. This timeframe also coincides with the implementation of regulatory limits on work hours established by the European Working Time Directive, which took effect in 1993. Some articles were identified during a search for specific authors considered experts within this domain of knowledge. Others were identified through citations and bibliographies of previously accessed professional/trade literature. Since a key objective of this review is to quantify the effects of excessive work hours to identify safe work hour limits, only quantitative research articles were considered. A sample search strategy is given in Table 1.

**Table 1:** Sample search strategy for a rapid review of the literature on the occupational health and safety effects of long work hours and work-related fatigue.

Databases	Search terms
Ovid MEDLINE	1. Safety-sensitive industr.mp or 24/7 operation.mp 2. long work hours.mp 3. extended work hours.mp 4. excessive work hours.mp 5. extended work shifts.mp 6. occupational fatigue.mp 7. work-related fatigue.mp 8. occupational health and safety.mp 9. occupational health.mp 10. (1) AND (2 OR 3 OR 4 OR 5 OR 6 OR 7) AND (8 OR 9)
1990 to present (June 2023)	

**Figure 1:**
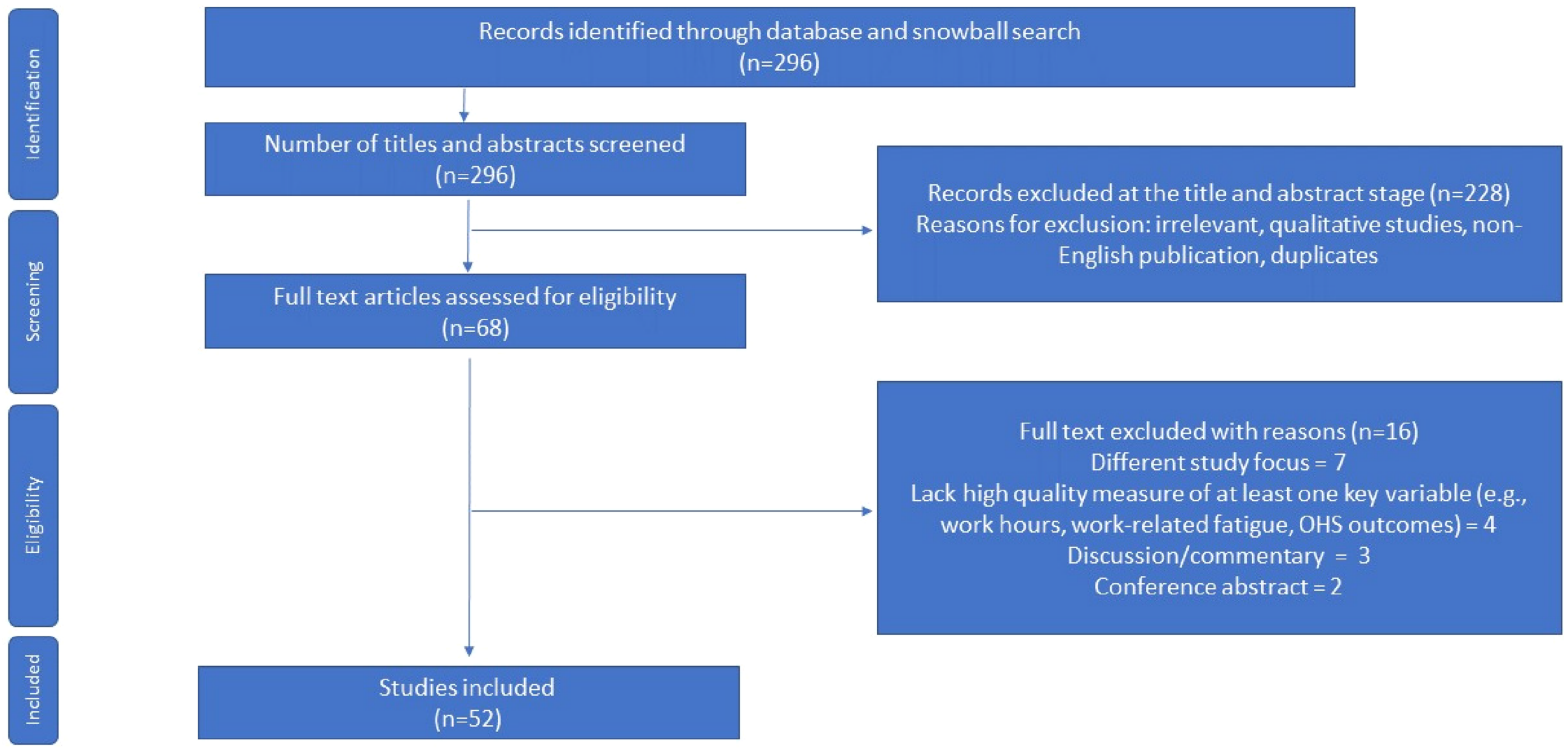
Modified PRISMA flow diagram of studies identified for rapid review.

The initial search, completed in June 2023, yielded 296 articles. Adjudication of articles for inclusion in the review was informed by the a modified version of the Preferred Reporting Items for Systematic Reviews and Meta-Analyses (PRISMA) for rapid reviews (13). Use of this framework allows for a coherent synthesis of knowledge based on a targeted research question coupled with search strategies that make it easier to identify relevant papers. In accordance with the requirements of a rapid review, however, components of the systematic review process were simplified or omitted to produce an evidence base in a timely manner (14). Figure 1 outlines the inclusion/exclusion criteria. In all, 52 studies were deemed both relevant and of sufficiently high quality for inclusion in the main review.

The identified studies fell into two categories. First, the human factors literature examines the etiology of human fatigue rooted in neurophysiological processes, including circadian function and the homeostatic drive for sleep. These studies provide a foundation for understanding how work hours are limited by human biology. The second category is epidemiological research on the occupational health and safety effects of excessive work hours and work-related fatigue within specific work contexts, with a focus on safety-sensitive industries, including health care. These studies consider the effects of real-world factors on fatigue-based risk *in situ*, with implications for occupational safety outcomes at the individual and organizational levels. An overview of the selected studies and the extracted evidence by category (author, year of publication, country, outcome variable, method, relevant findings) is provided in Table 2.

**Table 2:** Overview of selected studies on occupational health and safety outcomes of long work hours.

	Country/region	Outcome variable	Method	Relevant findings
Ariza-Monteset al. (15)	EUROPE	Workplace conflict and lateral violence	Survey of health care workers (*n* = 284)	Health care workers on rotating shift schedules were over twice as likely to report experiencing workplace bullying.
Artazcoz et al. (16)	CATALONIA	Mental and physical health indicators	Survey of salaried workers aged 16 to 64 (*n* = 7,103)	Gender stratified results showed that long work hours (>40 hours per week) was linked with poor mental health, hypertension, smoking, sleep shortage, and lack of leisure-time physical activity; among women, there was a heightened risk of smoking and sleep deprivation.
Baldwin & Daugherty (17)	US	Patient care and personal heath variables	Survey of medical residents (*n* = 2,813)	Working over 80 hours per week was strongly linked to residents feeling impaired by fatigue, experiencing conflict with colleagues, making medical errors, and having personal accident or injuries.
Barger et al. (18)	US	Motor vehicle crashes and near miss incidents	Survey of medical residents (*n* = 2,737)	The odds ratio for motor vehicle crash or a near-miss incident after an extended work shift (≥24 hours) was 2.3 and 5.9, respectively. Each extended shift that was scheduled in a month increased the montly risk of a crash during the commute from work by 16.2 percent.
Bonde et al. (19)	INTERNATIONAL	Breast cancer in women working night shifts	Narrative review of epidemiological and experimental studies (*n* = 12)	Women with previous or current breast cancer should be advised not to work night shifts because of strong experimental evidence demonstrating accelerated tumor growth by suppression of melatonin secretion
Bushnell et al. (20)	INTERNATIONAL	Health behaviours	Survey of manufacturing workers (*n* = 26,442)	Prevalence of unhealthy behaviours such as no exercise, obesity, smoking, and moderate to heavy alcohol consumption were significantly higher among individuals working extended shifts (≥12 hours) and/or night shifts.
De Raeve et al. (142)	NETHERLANDS	Interpersonal conflicts at work	Survey of workers in blue- and white-collar jobs (*n* = 9,241)	Overtime, shift work, and high physical demands were significant predictors of conflict with both coworkers and supervisors.
Dembe et al. (21)	US	Work injury	Survey of working-aged adults (*n* = 12,686); subsample of injuries among health care workers (*n* = 545)	Health care workers were at the highest risk of injury when working overtime or schedules of 60 hours or more per week (RR = 2.02) or worked more 12 hours per day (RR = 1.22).
Dembe et al. (143)	US	Work injury and illness	Survey of working-aged adults (*n* = 10,793)	After adjusting for age, gender, occupation, industry and region, jobs with overtime schedules were linked to a 61% higher risk of injury. Working 12 or more hours per day was associated with a 37% increase in injury risk, while working 60 hours per week was associated with a 23% increase in risk.
Dembe & Yao (22)	US	Chronic disease	Survey of working-aged adults (*n* = 7,492)	Longitudinal analyses conducted over 32 years (1979 to 2009) found that consistently working long hours significantly increased the risk of heart disease, non-skin cancer, arthritis, and diabetes, with the observed risk being notably higher among women.
Dong, X. (23)	US	Work injury	Survey of constuction workers (*n* = 10,840)	Long work hours and irregular work schedules were significantly associated with higher rates of work injuries after controlling for confounders.
Folkard, S. (24)	US	Work accident	Meta-analytic review of published trends (*n* = 6)	Collective evidence revealed a circadian rhythm in accident risk, with a major peak at 3am, coinciding with performance deficits during the window of circadian low. Additional spikes in accident risk were significantly influenced by shift duration, with individuals working 12-hour shifts facing an exponentially higher accident risk compared to those on shorter 8-hour shifts.
Folkard & Lombardi (25)	INTERNATIONAL	Work accident	Pooled risk estimates of published studies (*n* = 14)	Using the standard work week of five consecutive eight-hour day shifts with a single mid-shift break as a baseline, pooled risk estimates showed that four consecutive 12-hour day shifts increased the risk of accidents by 25%. An interaction effect related to shift timing showed that four consecutive night shifts raised the accident risk by 55%. Rest breaks taken every two hours were shown to significantly reduce safety risks.
Folkard & Tucker (26)	INTERNATIONAL	Work accident	Pooled risk estimates of published studies (*n* = 25)	The cumulative impact of consecutive shifts, combined with shift timing, leads to an increased overall risk of safety incidents. The likelihood of an incident on successive morning or day shifts rises by 2%, 7%, and 17% on the second, third, and fourth shifts, respectively. For night shifts, this risk increases more significantly, by 6%, 17%, and 36%, on the corresponding shifts.
Gander et al. (27)	NEW ZEALAND	Medical errors	Survey of physician anesthetists (*n* = 301)	Weekly work hours surpassed personal safety limits for 50% of trainees and 27% of specialists. Physicians who exceeded their self-identified safety thresholds were significantly more likely to report medical errors.
Geiger-Brown et al. (28)	US	Fatigue in nurses	Survey of female registered nurses (*n* = 175)	Nurses working extended shifts reported an average sleep duration of 5.5 hours between 12-hour shifts; nurses were progressively sleepier each shift, and night nurses were sleepier toward the end of their shift.
Gershon et al. (10)	US	Percutaneous injury in nurses	Survey of registered nurses (*n* = 738)	Mandatory overtime increased the risk of purcutaneous injury in nurses more than twofold (OR = 2.44).
Hänecke et al. (29)	GERMANY	Work accident	Workers compensation data of registered work accidents (*n* = 1.2 million)	An exponential risk curve was observed for work shifts longer than nine hours. A significant interaction effect was found between hours worked and time of day, with later start times linked to a sharp rise in accident risk after the 8th hour of work..
Heikkila, K., et al. (30)	DENMARK, GERMANY, FINLAND, SWEDEN, THE NETHERLANDS, UK	Cancer risk	Survey of workers who were cancer-free at baseline (*n* = 116,462)	Prospective analyses over 10.8 years identified a link between working more than 55 hours per week and a higher risk of breast cancer in women.
Hu et al. (31)	TAIPEI	Mental burnout	Survey of full-time employees (*n* = 1,560)	Extended working hours were found to predict burnout in a dose-dependent way. Those working over 40 hours per week had a burnout rate 58% higher than those on a standard schedule, while those working over 60 hours per week were 129% more likely to suffer from mental burnout.
James et al. (32)	US	Cognitive effectivness as a proxy for work performance	Nurses sleep/wake schedules monitored using wrist actigraph devices	Compared to day shift nurses, night shift nurses experienced more frequent and severe drops in cognitive effectiveness during their shifts, spending a significantly larger portion of their work time in the "high-risk zone" for performance errors.
Kang et al. (33)	INTERNATIONAL	Cardiovascular disease	Meta-analysis of published studies (*n* = 11)	The effect of long working hours (≥ 50 hours per week) was significantly associated with the risk of CVD in the random-effects model of all 11 studies (OR = 1.37)
Kivimäki et al. (34, 35)	INTERNATIONAL	Cardiovascular disease	Meta-analysis of published (*n* = 4) and unpublished studies (*n* = 19)	Working long hours (55 or more per week) was linked to a 29% higher risk of developing Type 2 diabetes. Stratified analyses indicated that this relationship was significant particularly among workers with lower household incomes
Kleppa et al. (36)	NORWAY	Mental health outcomes	Survey of overtime workers (*n* = 1,350) compared to reference group (*n* = 9,092)	Comparative analyses revealed that workers with overtime schedules had significantly higher rates and more severe cases of anxiety and depression compared to those on standard schedules. A dose-response relationship was also observed, with increased work hours correlating with greater levels of anxiety and depression.
Ku & Smith (37)	US	Fatigue, social wellbeing, interpersonal conflict	Survey of locomotive engineers and conductors (*n* = 125)	Organizational factors, such as work scheduling and the number of work hours, were significantly linked to an increased likelihood of interpersonal conflict among coworkers. Social well-being played a mediating role, helping to reduce the impact of scheduling factors on inter-worker conflict.
Landrigan et al. (38)	US	Medical errors	Comparative analysis of medical errors by work schedule within a sample *n* = 2,203 patient-days	Interns following a traditional schedule with extended shifts slept an average of 5.8 hours less per week and made 35.9% more serious medical errors. Extended hours were associated with a 20% higher rate of medication errors and a sixfold increase in diagnostic errors.
Lo et al. (11)	TAIWAN	Needlestick and sharp injuries	Survey of full-time bedside nurses (*n* = 19,386)	A dose-response relationship was observed between work hours and the risk of injury. Nurses working 41 to 50 hours per week were 1.17 times more likely to sustain a sharps injury, while those working more than 50 hours per week had a 1.51 times greater risk.
Matre et al. (39)	INTERNATIONAL	Work safety incidents	Meta-analysis of published studies (*n* = 22)	Results showed a significant association between working >12 hours/day, or working > 55 hours/week and elevated risk of safety incidents (RR = 1.24 for each overtime category, respectively)
McCormick et al. (40)	US	Medical errors	Monitoring of medical residents' sleep/wake cycles via actigraphy (*n* = 128)	Residents working night shifts were impaired by fatigue for nearly half of their working hours and had an average effectiveness score of 70, representing a FE-BAC of 0.05%. Fatigue countermeasures such as 30-minute naps and replacing 24-hour shifts with 12-hour shifts, proved highly effective, reducing the percentage of waking time residents were impaired by fatigue to below 2 percent.
McCormick et al. (144)	US	Work performance/task effectiveness, medical errors	Monitoring of medical residents' sleep/wake cycles via actigraphy (*n* = 27)	Residents experienced fatigue during nearly half of their waking hours, with critical impairment (equivalent to a blood alcohol concentration of 0.08%) occurring for over one-quarter of that time. The predicted impact of this fatigue was a 22% increase in the risk of medical errors.
Megdal et al. (41)	INTERNATIONAL	Breast cancer	Meta-analytic review of published studies (*n* = 13)	Working night shifts was associated with a 48% increase in breast cancer risk among female night workers.
Muecke, S. (42)	INTERNATIONAL	Health outcomes	Rapid review of published studies (*n* = 29)	Researchers agree that night rotations have detrimental psychological and physiological effects on nurses, with these impacts being more pronounced in nurses over the age of 40.
Olds & Clarke (8)	US	Needlestick and other work-related injuries, medical errors	Survey of registered nurses (*n* = 11,516)	Working more than 40 hours per week was associated with a 28% higher risk of medication errors and needlestick injuries. Overtime hours raised the risk of these incidents by 20% and 30%, respectively.
Palancı et al. (43)	TURKEY	Workplace conflict	Survey of health care workers (*n* = 708)	Poor working conditions and high levels of work-related stress were significantly linked to an increased likelihood of conflicts with colleagues. This relationship was more pronounced among individuals working more than 16 hours of overtime per week.
Pogue et al. (44)	US	Workplace bullying	Survey of nurses (*n* = 943)	Nurses were more likely to experience workplace bullying if they worked longer weekly hours or had higher amounts of overtime.
Rivera et al. (45)	INTERNATIONAL	Chronic conditions	Systematic review of SRs with meta-analyses (*n* = 48)	Shift work and long work hours were linked to the onset of several chronic conditions, including cardiovascular disease, cancer and depression.
Rodrigues et al. (46)	BRAZIL	Fatigue risk	Work schedule and sleep/wake data for pilots and aircrew (*n* = 8,476)	Biomathematical modeling revealed that fatigue risk increases linearly with the number of consecutive night shifts. An exponential relationship was observed between fatigue hazard and the number of critical flight phases occurring during the circadian low window (between 2 a.m. and 6 a.m.). Fatigue countermeasures, such as afternoon naps, reduced overall fatigue hazard during critical flight phases from 63% to 43%.
Rogers et al. (47)	US	Medical errors	Survey of nurses (*n* = 393)	Shift duration, overtime hours, and total weekly hours had significant impacts on error frequency. Nurses working shifts of 12.5 hours or more were over three times more likely to make a medical error (OR = 3.29). Overtime work increased the odds of errors regardless of the originally scheduled shift length (OR = 2.1). Additionally, nurses working more than 40 hours per week faced nearly double the risk of making medical errors (OR = 1.96).
Sagherian et al. (48)	US	Work absence	Monitoring of nurses' work-rest shedules and work absence data (*n* = 197)	Biomathematical modeling showed that one in four 12 hour shifts were worked by nurses experiencing high levels of fatigue (FAID score ≥ 80, FE-BAC = 0.05%). High FAID scores were associated with a greater propensity for work absence.
Schwartz et al. (145)	US	Work performance	Analysis of work schedule data among medical residents (*n* = 89)	Performance scores declined as shift length increased; after 16 hours, residents spent 29% of their shift functioning below the critical effectiveness threshold, equivalent to a FE-BAC of 0.05%. Ninety-minute naps significantly reduced the percentage of time residents operated below this effectiveness level.
Scott et al. (12)	US	Drowsy driving	Survey of shift-working nurses (*n* = 895)	Two-thirds of nurses reported experiencing at least one episode of drowsy driving (either a motor vehicle crash or a near miss) during a four-week observation period. Nurses working shifts of 12.5 hours or longer had twice the risk of drowsy driving compared to those working 8.5 hours or less. The risk was significantly higher for those working night shifts.
Shields, M. (49)	CANADA	Health behaviours	Survey of Canadian workers (*n* = 17,626)	Men working more than 40 hours per week had twice the risk of smoking, while women had a fourfold increased risk. Women working long hours also had significantly higher rates of daily alcohol consumption. Additionally, men faced a greater risk of having an unhealthy BMI.
Thompson, B. (50)	US	Physiology-based performance outcomes	Physiological measures in nurses (*n* = 26)	Work-induced fatigue was found to accumulate over 12-hour shifts. Psychomotor vigilance reaction time and lapses of attention significantly decreased from the end of shift one to the end of shift three. Muscle function variables also showed declines after just a single work shift.
Trépanier et al. (51)	CANADA	Bullying behaviours	Survey of nurses (*n* = 275)	Controlling for baseline exposure to bullying behavior, workload and time pressures in nurses working more than 10 consecutive hours were predictive of increased exposure to bullying behavior, particularly when job recognition and social support were low.
Trinkoff et al. (9)	US	Patient mortality	Survey of hospital nurses (*n* = 633)	After adjusting for staffing characteristics and hospital attributes, nurses' work schedule was significantly related to mortality outcomes. Pneumonia deaths were significantly more likely in hospitals where nurses reported long work hours and insufficient time away from work (OR = 1.42 and 1.24, respectively). Mortality from acute myocardial infarction was associated with weekly hours burden, including hours worked per week and consecutive working days (OR = 1.33).
Virtanen et al. (52)	UK	Mental health outcomes	Survey of full-time civil service employees (*n* = 2,960)	Hazard analyses adjusting for baseline covariates revealed a 1.66-fold increase in the risk of depressive symptoms and a 1.74-fold increase in the risk of anxiety symptoms among employees working more than 55 hours per week. Sex-stratified analyses indicated that the excess risk of depression and anxiety associated with long working hours was particularly pronounced in women (OR = 2.67).
Virtanen et al. (53)	INTERNATIONAL	Coronary heart disease and stroke	Systematic review and meta-analysis of published and unpublished studies (*n* = 42)	Working long hours (55 or more per week) was associated with an increased risk of coronary heart disease and stroke (RR = 1.13 and 1.33, respectively). A dose-response relationship was observed for stroke, with risk estimates of RR = 1.10 for 41-48 working hours, RR = 1.27 for 49-54 working hours, and RR = 1.33 for 55 or more hours per week.
Wagstaff & Sigstad Lie (54)	INTERNATIONAL	Work safety outcomes	Rapid review of published studies (*n* = 14)	Work periods exceeding 8 hours are associated with a cumulative increase in accident risk, with the risk at 12 hours being double that at 8 hours. Shift work, particularly involving night shifts, adds a significant additional risk for workplace safety incidents.
Watanabe et al. (55)	INTERNATIONAL	Metabolic syndrome	Meta-analysis of published studies (*n* = 8)	Work related psychosocial factors, including long work hours and shift work,
Wolf et al. (56)	US	Workplace aggression	Survey of ER nurses	Fatigue from shift timing and long hours was a significant determinant of a toxic work culture and incidents of lateral violence among ER nurses.
Wong et al. (57)	INTERNATIONAL	Occupational health outcomes	Meta-analysis of published studies (*n* = 48)	Long work hours (>10 hours per day or >50 hours per week) were associated with elevated risk of cardiovascular disease (OR = 1.54) and metabolic syndrome (OR = 1.10).
Yinghui et al. (58)	US, JAPAN, TAIWAN	Patient safety outcomes	Survey of nurses in three countries (US = 106,710, Japan = 4,407, Taiwan = 5,714)	In all three countries, nurses working 40 or more hours per week were more likely to report patient-related safety events. In Japan and the US, overall scores for patient safety grade were significantly lower among nurses working 60 or more hours per week.

### 2.2 Human factors research on the etiology of occupational fatigue

Human factors research is a broad multidisciplinary field that focuses on understanding and optimizing the interaction between humans and aspects of their environment. With respect to work and workplace settings, the goal is to develop work systems that enhance human well-being, performance and satisfaction while minimizing errors, accidents, and adverse effects (59).

A key area of human factors research is understanding how to minimize the onset and consequences of work-related fatigue. Broadly, fatigue at work arises from an imbalance between the intensity, duration, and timing of work, coupled with an insufficient allotment of time for recovery (60). In real-life work environments, this imbalance is linked to poor scheduling practices that require workers to stay on task for extended periods without sufficient rest-breaks either during or between shifts, and is compounded by high-intensity workloads. From an occupational standpoint, fatigue is commonly described as an individualized experience of lack of energy or tiredness with physical, cognitive and/or psychological manifestations (48). Work-related fatigue is a significant workplace hazard since it leads to diminished cognitive and physical acuity, which raises the risk of safety incidences on the job (25, 61–63). While there are different dimensions of fatigue (e.g., muscular, mental, psychomotor), human factors studies primarily focus on fatigue related to the “drive to sleep,” which stems from the neurobiological processes regulating sleep and circadian rhythm (64). Sleep is essential to recovery after prolonged work-related activity. Its functional benefits include restoration and repair of both physical and cognitive systems, conserving energy, and strengthening the immune system. In the short term, sleep deprivation leads to reduced attention, slower reaction times, impaired memory consolidation, and diminished emotional regulation (65). Over the long term, accumulating sleep deficits are linked to declines in physical and mental health, contributing chronic conditions such as cardiovascular disease, diabetes, and depression (66, 67).

Bio-mathematical models of fatigue (BMMF) have been used to isolate the basic human factors that give rise to fatigue among workers across a range of settings. The models are designed around assumptions regarding the interaction of basic neurobiological functions, namely homeostatic drive for sleep and processes of circadian regulation (61). Drawing on information related to work-rest patterns, BMMF constitute predictive tools with respect to the level of fatigue associated with different work schedules, and are able to quantify the inferred risk on performance and safety outcomes arising from fatigue (60).

Several studies have used BMMF to evaluate the degree of “hazard exposure” associated with work-related fatigue. These studies quantify the relative risk arising from fatigue across a range of work practices and industry settings. Within health care, studies have used bio-mathematical modeling to examine the association between long hours, fatigue and fatigue-related risks (e.g., medical errors). Sagherian et al. (48) used FAID (Fatigue Audit Inter Dyne) to estimate fatigue in pediatric nurses. FAID scales are calibrated such that a fatigue score of 40 represents a standard work schedule of 40 h per week, Monday to Friday 9:00 a.m. to 5:00 p.m. Scores of 80 to 100 represent work-related fatigue arising from 23 to 24 h of continuous sleep deprivation; scores higher than 120 represent a permanent night shift schedule of six consecutive 12 h night shifts, with one day off between shifts. Nurses working 12 h shifts generated FAID scores of seven to 154 with nearly one in four (23%) shifts worked by nurses experiencing very high levels of fatigue [i.e., FAID scores of 80 or more indicating a Fatigue Equivalent BAC (FE-BAC) of 0.05%]. James et al. (32) used another BMMF approach known as SAFTE (Sleep Activity Fatigue Task Effectiveness) to analyze nurses’ sleep/wake schedules. Significant differences in cognitive effectiveness between shift-type were found with night shift nurses exhibiting frequent substantial declines into the “high risk” zone throughout their shifts (32).

Schwartz et al. (145) examined the effects of duty-hours on performance in surgical residents using SAFTE software to predict fatigue risk and performance outcomes. Performance scores decreased with increased shift length; after 16 h, residents spent over one-fourth of their shift (29%) below the “critical effectiveness threshold” (FE-BAC = 0.05%). Additional analyses found that naps lasting 90 min significantly reduced the predicted percentage of time residents spent below the critical threshold. Similar studies study by McCormick et al. (40, 144) found that medical residents experience fatigue nearly half (48%) of their waking hours, with their fatigue levels reaching the “impaired” threshold (FE-BAC of 0.08%) 27% of the time (144). Fatigue in residents was associated with a 22% increased risk of medical errors. Additionally, residents working night shifts were more fatigued and faced a greater risk of medical errors compared to those on day shifts. Fatigue countermeasures such as 30 min naps and substituting 24 h shifts with 12 h shifts were found to be highly effective, reducing the percentage of waking time residents were impaired by fatigue to below 2 percent (40). Other studies have demonstrated the risk-mitigating effects of limiting the number of critical work tasks performed during the window of circadian low (WOCL), which occurs between about 2 a.m. and 6 a.m. (46).

### 2.3 Epidemiological studies on the health and safety risks of long work hours

A substantial body of epidemiological evidence demonstrates the risks associated with long work hours across various work contexts. In general, these studies are concerned with fatigue resulting from sleep deprivation caused by work scheduling factors, such as overtime and extended shifts lasting more than eight hours. Studies tend to focus on the impacts of occupational fatigue within safety-critical industries such as aviation, transportation, heavy manufacturing and health care. Focal outcomes generally fall into three categories: (1) the risk of workplace safety incidents (i.e., accidents, including errors, or injuries); (2) the risk of workplace conflict and lateral violence; and (3) risks to individual health and wellbeing.

#### 2.3.1 Long hours and the risk of workplace safety incidents

The elevated risk of a workplace safety incident can result from a combination of total successive work hours coupled with shift timing. Several high-profile industrial disasters have been partly attributed to occupational fatigue from long nighttime hours, including the Exxon Valdez oil spill disaster, the Challenger Space Shuttle explosion, and system failures at Three Mile Island and Chernobyl (68–70). In health care, Canadian data show that one in 17 hospital stays involved at least one harmful event (71). A portion of iatrogenic incidents may be due to work hours and fatigue among health care providers (72).

Research on the impact of prolonged work hours and the timing of work has quantified the effects of fatigue and associated workplace risks. A notable early study by Folkard (24) examined the effects of shift timing and duration on the risk of accident in transport operations. Sleep propensity data identified peak accident risk occurs at 3am due to lower performance during the window of circadian low. Time-on-task was also a significant factor with shifts lasting 12 h or more at twice the accident risk of 8 h shifts (24). Other studies have reported similar findings with respect to the exponential risk of accident beyond the 9th hour of work. Using a sample of 1.2 million registered workplace accidents, Hänecke et al. (29) reported a significant interaction between hour at work and time of day with later start times predictive of a drastic increase in accident risk beyond the eighth hour at work (29).

A prospective study drawing on a large representative panel of US workers found that the odds of injury after 12 h of work nearly doubled, while shifts lasting longer than 16 h produced a hazard rate of 3.5. Workweeks of ≥50 h nearly doubled the risk of workplace accident (OR = 1.98) (23). In another analysis of the same panel, Dembe et al. (143) observed that, after controlling for factors like age, gender, occupation, industry and region, jobs involving overtime schedules were associated with a 61% higher injury risk. Furthermore, working 12 or more hours per day was increased injury risk by 37%, while working 60 h per week elevated the risk by 23% (143). Another study involving health care professionals demonstrated a clear dose-response relationship between weekly work hours and risk of injury. No additional risk was found to be associated with extended shifts lasting 12 h. The authors concluded that the most potent source of fatigue in health care workers arises from accumulated hours over the course of a week, rather than from occasional 12 h shifts worked a few days each week (21).

**Figure 2:**
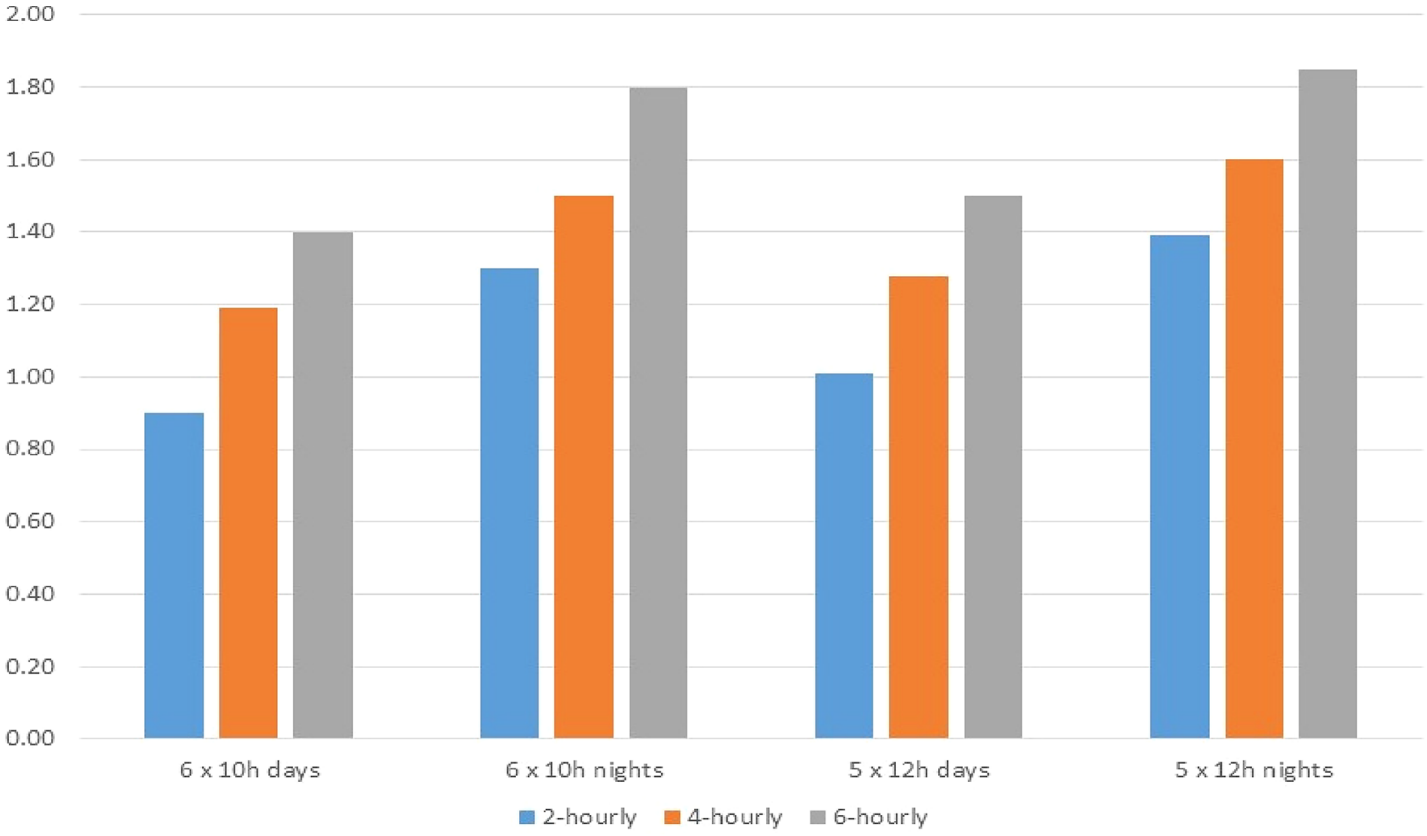
Risk of accident by type of work shift including rest breaks. Source: Folkard & Lombardi (25).

Folkard and Lombardi (25) employed an advanced analytic approach to investigating the risk of long work hours by pooling data across several studies (25). Pooled risk estimates were used to construct a “Risk Index” based on different combinations of aspects of work scheduling—i.e., shift duration, number of successive shifts, shift timing, intervals between breaks. Using the “normal work week” comprised of five successive eight-hour day shifts with a single mid-shift break as a baseline, the study found that four successive 12 h day shifts increased accident risk by 25%. There was an interaction effect for shift-timing with four successive night shifts increasing accident risk by 55%. The authors concluded that work schedules should follow the general guideline that for any given workweek, a long span of short shifts tends to be safer than a short span of long shifts (e.g., six eight-hour shifts vs. four consecutive 12 h shifts). Furthermore, properly timed rest breaks—ideally every two-hours—can significantly reduce safety risk (Figure 2).

Folkard and Tucker (26) reported similar findings regarding the cumulative effects of multiple shifts and shift timing showing that, on average, the risk of incident on successive morning/day shifts is 2%, 7% and 17% higher for the second, third and fourth work shift respectively, whereas for night shifts the respective risk is elevated to 6%, 17% and 36% (26). Furthermore, at least two systematic reviews provide strong evidence of the cumulative safety risk associated with long work hours. The risk of safety incident for shifts lasting 12 h can be as high as twice that of shifts lasting eight hours (39, 54). Among nurses, Thompson (50) has shown that extended 12 h work shifts lasting 12 h were linked to reduced muscle strength, slower reaction time and decreased attention. Additionally, both reaction time and attention continued to deteriorate over successive work shifts (50).

Other studies have confirmed the relationship between long work hours and safety risk with respect to the commission of medical errors. Gander, Merry, Millar, & Weller (27) has reported that physicians who exceed self-defined safety limits for consecutive work hours were significantly more likely to report medical errors (27). Landrigan et al. (38) used a prospective, randomized design to investigate the impact of long hours on medical interns in critical care units. Interns following a traditional schedule with extended shifts slept an average of 5.8 h less per week and made serious medical errors 35.9% often compared to the intervention group, which eliminated extended shifts and reduced weekly work hours. Additionally, extended hours were linked with a 20% higher rate of medication errors and a six fold increase in serious diagnostic errors (38). Rogers et al. (47) investigated nursing care outcomes in relation to work hours and shift timing. Shift duration and number of hours worked per week had a significant effect on error frequency. Shifts of 12.5 h or more were associated with an error rate more than times that of eight-hour shifts. Overtime hours were predictive of a two-fold increase in the odds of making at least one error. Dose-response between work hours and incident risk was reported with shifts of 8 h, 8–12 h and 12 h increasing error risk by 34%, 53% and 326%, respectively. Nurses working more than 40 h per week were at nearly twice the risk of patient errors and at almost 50% higher risk of near errors (47). Another study of the effects of extended work hours on critical care nurses reported similar findings with shifts lasting 12.5 predictive of nearly twice the probability of medical error occurrence (7). The probability of near errors also increased significantly during extended shifts. Nurses working more than 40 h per week were at significantly higher risk of both errors and near errors (46% and 93%, respectively). At least one study has directly linked long work hours in nurses with mortality outcomes in patients (9). Adjusting for staffing (e.g., levels and skill mix) and hospital characteristics, work schedule was found to be significantly associated with mortality outcomes. Deaths from pneumonia were 42% more likely in hospitals where nurses worked extended hours and lacked time away from work. Mortality from acute myocardial infarction was significantly associated with weekly work hours burden. A review by Muecke (42) examining 10-years of scientific literature on the effects of rotating night shifts in nurses reported evidence of a relationship between extended shifts, inferior levels of alertness and performance, failures in attending to changes in patient condition, medication errors, and patient mortality (42).

Research has shown that nurses working extended hours face a heightened risk of work-related injuries. In an analysis of reports from over 11,000 nurses, Olds and Clarke found that both needlestick injuries and medication errors were 28% more common among nurses working more than 40 h per week. Overtime hours also increased the risk of needlestick injuries by 20% and medication errors by 30% (8). Another study involving over 19,000 bedside nurses revealed that those working more than 40 h per week were 17% more likely to experience needlestick injuries and 29% more likely to suffer sharps injuries. For nurses working over 50 h per week, these risks increased to 51% and 37%, respectively (11).

Studies have also demonstrated that the risks associated with long work hours are not constrained to accidents at the workplace. Several studies have demonstrated the risks of post-shift fatigue on drowsy driving and motor vehicle accidents (18, 73, 74). Scott et al. (12) examined the effects of shift work on drowsy driving in nurses. Two-thirds of nurses reported at least one episode of drowsy driving; ∼5% of nurses reported struggling to stay awake while driving home after every shift. The risk of reporting a drowsy driving episode doubled among nurses working 12.5 h or more. Night shifts nurses reported difficulty staying awake driving home 30% of post-shift periods (12).

#### 2.3.2 Long hours and the risk of workplace conflict and lateral violence

Long work hours have been linked with mental and emotional exhaustion, increasing the likelihood of workplace conflict. In nursing, excessive work hours are a critical factor within a broader set of work-related conditions—such as high intensity work, heavy workloads, and high stakes outcomes—that are strongly predictive of elevated stress and burnout (75, 76). These stressors can significantly influence the organizational climate, creating an environment that fosters interpersonal conflict and psychological bullying (77, 78). Studies of workplace conflict in health care workers have found factors including shift work, long work hours, high stress work settings, heavy workloads, and poor working conditions are associated with inter-peer conflict and lateral violence (15, 51, 79, 142). Excessive work hours tend to increase stress and fatigue, causing breakdowns in communications among staff, reduced empathy and patience for the collaborative needs of others, and an increase in general irritability and conflict triggers (37, 80).

Studies have shown that the poor working conditions of nurses can deplete mental and emotional resources, making them more prone to negative mood (51, 81). Collectively, the impoverished psycho-social conditions of the work environment can significantly diminish the quality of the relationships within nursing units and increase the likelihood of hostile and abusive behaviour among staff (51, 58). One study found that average scores for teamwork, based on supportiveness, respect and cooperation, were significantly lower among nurses working longer hours (≥60 h per week), compared to those working <40 h per week. Other studies have demonstrated similar effects of long hours and overtime on occupational stress and the tendency for workplace conflict (43, 56). The degradation of key aspects of teamwork—such as treating coworkers with empathy and respect, and demonstrating a willingness to collaborate on tasks—constitutes a crucial leading indicator of a potential breakdown in healthy work relations, with implications for the quality of nursing care and patient safety (17, 43, 58, 82).

There is a growing body of evidence that a lack of control over work schedule coupled with high workloads can produce a work climate wherein lateral violence, or bullying, among nurses can flourish (77, 83, 84). Trépanier et al. (51) followed nurses over one-year to examine the effects of workload and work-related time pressures on bullying incidents. Controlling for frequency of bullying encounters at baseline, workload was found to be positively predictive of exposure to bullying behaviours over time when both job recognition (i.e., perceived social rewards from others at work) and social support were low (51). A recent study by Pogue and colleagues found that that nurses were more likely to experience workplace bullying the more hours they worked per week. Nurses who experienced bullying were significantly less likely to report good or excellent quality of care within their units, as well as a favourable grade for patient safety (44).

#### 2.3.3 Long hours and the risks to individual health and wellbeing

Long hours and compressed work schedules are common in nursing; still, accumulating evidence indicates the potential negative effects on both mental and physical well-being resulting from these scheduling practices. Extensive epidemiological studies have established associations between non-traditional work hours, such as extended shifts and shift work, and the increased risk of various chronic health issues.

##### 2.3.3.1 Sleep disorders

Long work hours and overtime diminish the available time for sleep, leading to fatigue (85). Among shift workers, quantity of sleep can be reduced by up to two hours a day; moreover, the quality of sleep tends to be diminished due to a lack of REM and Stage 2 sleep (86). Evidence suggests that few shift-working nurses obtain the recommended seven to eight hours of sleep during recovery periods (87). Self-reported sleep times range between 4.3 and 6.7 h, with night shift nurses generally getting less sleep than their daytime counterparts, averaging 5.7 h compared to 6.7 h, respectively (88). Consequently, night-shift nurses typically report heightened levels of fatigue and can are more susceptible to cumulative adverse effects (89). An investigation of sleep habits among nurses working extended shifts found that sleep duration averaged 5.5 h between 12 h shifts (28). Other key studies on the impacts of shift work among nurses have shown that sleep deficits resulting from extended work hours can induce sleepiness during shifts and increase the likelihood of involuntary napping, both of which are associated with a higher risk of medical errors (7). Over time, disordered sleep patterns can lead to chronic insomnia, a significant problem among shift-working nurses (11).

##### 2.3.3.2 Mood disorders

A growing body of evidence has established a connection between extended work hours and the development of mood disorders. Research findings from this literature indicate that individuals working more than the standard 40 h per week are at an elevated risk of experiencing anxiety and depression (45, 52, 90). One longitudinal study of the psychological impacts of long work hours, utilizing an exceptionally robust sample of 2,960 workers, examined outcomes over a five-year period (52). Adjusting for baseline covariates, hazard analyses revealed a 1.66-fold increase in the risk of depressive symptoms, and a 1.74-fold increase in risk of anxiety symptoms among employees working more than 55 h per week, compared to those working 35–40 h per week. Sex-stratified analyses showed that the excess risk of depression and anxiety due to long work hours was especially pronounced in women (OR = 2.67) (52). Kleppa et al. (36) have reported similar results. Findings from comparative analyses showed that individuals who worked overtime hours exhibited both a higher prevalence and a greater severity of anxiety and depressive symptoms relative to those working standard hours. A dose-response relationship was also evident, with more work hours associated with higher levels of anxiety and depression (36). Hu et al. (31) have reported a dose-dependent association between long working hours and mental burnout. The odds of burnout was significantly greater in individuals working over 40 h per week (OR = 1.58). The odds of burnout substantially higher among those working more than 60 h per week (OR = 2.29). The authors recommend policies restricting weekly work hours to 40 to help prevent the onset of burnout (31).

##### 2.3.3.3 Cardiovascular disease

Many studies have documented the relationship between long work hours and an increased risk of cardiovascular disease. Rivera et al. (45) conducted a review of 48 individual systematic reviews investigating the links between work schedule and several chronic conditions. The review found low grade evidence that long work hours and shift work are associated with low to moderate increases in the risk of cardiovascular disease (45). A meta-analytic review by Kivimäki et al. (34, 35), using data from over 600,000 participants in the US, Europe, and Australia, reported similar findings. After adjusting for age, sex, and socio-economic status, long work hours (≥55 h per week) was predictive of an increased risk of coronary heart disease (RR = 1.13) and incident stroke (RR = 1.33) (34). Convergent conclusions were reached by at least two earlier meta-analytic reviews. Using results from 42 observational studies, Virtanen et al. (53) found that working long hours (>50 h per week) is associated with a 40% increased risk of coronary heart disease (53). Similarly, Kang et al. (33) analyzed evidence from 11 separate studies, with follow-up periods ranging from 42 months to 30 years on average, and concluded that individuals working long hours (≥50 per week) had a 37% higher risk of developing cardiovascular disease (33). Dembe & Yao (22) utilized data from an unusually large cohort of participants (*n* = 7,492) to investigate chronic disease outcomes over a period of more than three decades. The authors identified a dose-response relationship between average weekly work hours and reported prevalence of heart disease. Compared to those working the standard 40 h per week, those working 51 to 60 h had a 1.68 times higher risk, while working more than 60 h per week increased the risk by 1.74 times (22).

##### 2.3.3.4 Cancer

Several studies have linked long work hours and shift work with various types of cancer. In their longitudinal study tracking health outcomes in workers over three decades, Dembe & Yao (22) found that long work hours were associated with an elevated risk of non-skin cancers. Moreover, the relationship was cumulative, with individuals working 51 to 60 h per week experiencing a two-fold increase in the likelihood of cancer diagnosis (OR = 2.03), and workers exposed to more than 60 h per week exhibiting a risk increase of nearly three-fold (OR = 2.83). A multi-cohort study by Heikkila et al. (30) investigated the effects of excess work hours and shift work on incident cancer risk among workers who were cancer-free at baseline. Drawing on data from 12 separate prospective studies, the authors employed random effects meta-analysis to examine cancer rates among a total of 116,462 workers over an average period of 10.8 years. While the study did not show clear evidence of an association between working hours and overall cancer risk, the authors did find an association between working ≥55 h per week and an increased risk of female breast cancer (OR = 1.60) (30). Another meta-analytic review by Megdal et al. (41) found that exposure to night work was associated with a 48% increased risk of developing breast cancer (41). Bonde et al. (19) has also reported that statistically significant increases in risk for breast cancer were seen in people working night shift for 20 years or more; in turn, they conclude that women with previous or current be advised not to work night shifts because of strong experimental evidence demonstrating accelerated tumor growth by suppression of melatonin secretion (19).

In 2007, the International Agency for Research on Cancer (IARC) of the World Health Organization declared that there is sufficient evidence to support that shift work is a probable carcinogen. This designation is due to the disruption of the body's circadian rhythm caused by shiftwork (91). In their analysis of 17 systematic reviews on cancer outcomes in individuals working non-standard schedules, Rivera et al. (45) found moderate grade evidence linking shift work with female breast cancer.

##### 2.3.3.5 Poor health behaviours and metabolic syndrome

Research indicates that long work hours and shift work are associated with an increased risk of deleterious health behaviours. Bushnell et al. (20) tracked the effects of different types of work schedules on the rate of poor health behaviours over a period of nine years. After adjusting for potential confounders, the authors found that, compared to a standard 8 h day shift, a 12 h rotation schedule was predictive of higher rates of smoking (RR = 1.60), lack of physical activity and exercise (RR = 1.30), and short sleep duration of six hours or less per night (RR = 1.30) (20).

Artazcoz et al. (16) examined the relationship between weekly work hours and health-related behaviours among more than 7,000 salaried workers aged 16–64. Gender-stratified findings revealed that, among men, working 51–60 h per week was predictive of an elevated risk of hypertension (OR = 1.60), smoking (OR = 1.33), shortage of sleep (OR = 1.42), and no leisure-time physical activity (OR = 2.43). Among women, long work hours were associated with an increased risk of shortage of sleep (OR = 2.21) (16). Other studies have demonstrated an elevated risk of smoking in both sexes as a result of long work hours. Shields (49) has reported that, for both sexes, changing from standard work hours to long work hours (>40 h per week) is associated with increases in smoking behaviour. Among men, long work hours were predictive of more than twice the odds of increased daily smoking, while among women, the corresponding odds were more than four times higher (49).

Metabolic syndrome and its attendant risk factors have also been linked to work schedules. Shields (49) has reported that, among men, increased work hours are associated with unhealthy weight gain (49). Other studies have likewise linked increases in body mass index to shift work and excessive work hours (92, 93). A meta-analysis on the health effects of work-related factors by Watanabe et al. (55) demonstrated a significant positive relationship between shift work and metabolic syndrome (RR = 1.59) (55). A meta-analysis by Wong et al. (57) reported an association between long work hours and a heightened risk of metabolic syndrome (OR = 1.10) (57).

A handful of studies have identified work schedule as a risk factor in the onset of Type 2 diabetes. In a meta-analysis of data from more than 200,000 participants, Kivimäki et al. (34, 35) found that long work hours (≥55 h per week) were associated with an elevated risk of a Type 2 diabetes diagnosis in lower income populations (RR = 1.29) (35). Dembe & Yao (22) have reported that exposure to excessive work hours is predictive of an elevated risk of diabetes onset (22). Possible mechanisms underlying this relationship may include poor sleep quality and reduced sleep duration, which can cause an imbalance in appetite hormones, increasing feelings of hunger. These factors can lead to metabolic changes, resulting in obesity, insulin resistance and reduced lipid tolerance (94).

##### 2.3.3.6 Individual and organization-level factors affecting fatigue risk

Empirically-established thresholds regarding the effects of working hours on fatigue offer essential guidance for constructing safety limits. However, various individual and organizational factors can exacerbate occupational fatigue, thereby accelerating fatigue-based risk. At the individual level, factors influencing the onset of occupational fatigue include age, biological sex, health status, sleep habits, and personal circumstances that can affect opportunities for sleep and recovery, such as caregiving responsibilities. Within organizations, the structure of work systems can contribute to work-related fatigue and fatigue-related risks, including factors such as working conditions, heavy workloads, and work schedules that do not allow for adequate recovery time between shifts.

###### 2.3.3.6.1 Age

As individuals age, sleep patterns often change, characterized by shorter and more fragmented periods of sleep, with insomnia becoming more common (85, 95). Consequently, older workers may find it increasingly challenging to attain adequate recovery between work shifts (96, 97). Research on sleep deprivation indicates that performance deficits equivalent to a blood alcohol concentration (BAC) of up to 0.10% can occur, with more mature nurses reaching this performance limit in less time compared to their younger colleagues (98). Additional studies indicate that the range of adverse physical and psychological effects resulting from shiftwork-related fatigue is more pronounced in nurses over the age of 40 (42). While certain research suggests potential protective effects of experience and enhanced safety performance associated with older age and longer work tenure (99), it is important to note that the cumulative effects of fatigue stemming from shift work and extended work hours might counterbalance any incremental benefits linked with age (100, 101).

###### 2.3.3.6.2 Biological sex

Some evidence suggests that hormonal differences and gender-specific health issues can influence fatigue-levels, leading to differences in the ability of men and women to tolerate long hours and shift work. Studies examining the sex-stratified effects of non-standard work schedules have found that women working shift work or long hours experience shorter and lower quality sleep periods compared to their male counterparts (16, 96, 102, 103). Long work hours have been associated with a greater likelihood of the onset of depressive symptoms (49, 52, 104) and diabetes (105) in women, though at least one study has reported that adverse mental and physical outcomes resulting from excessive work hours tend to be more pronounced in men (16). Weekly work hours have also been demonstrated to have a stronger association with women's risk of work injury (106, 107). Some of the differences in susceptibility to the effects of extended work hours between women and men likely arise from disparities in childcare, other caregiving responsibilities, and domestic workloads, which disproportionately limit the amount of time women can dedicate to sleep and recovery from work (108).

###### 2.3.3.6.3 Health status

Research has demonstrated that certain chronic diseases can contribute to daytime sleepiness and compromise cognitive functioning. Chronic conditions known to impact fatigue include arthritis, asthma, chronic fatigue syndrome, chronic obstructive pulmonary disease (COPD), and rhinitis (109). As the population ages, the incidence of chronic morbidity due to various health conditions has increased. Currently, approximately one in three Canadians (33.7%) lives with a chronic condition, including cardiovascular disease, cancer, diabetes, or mood and/or anxiety disorders (110). The physical and psychosocial stresses linked with non-standard work schedules are known to be less well tolerated by individuals with chronic health issues (96, 108). One study observed that night shift workers with Type 2 diabetes had greater difficulty controlling glucose levels compared to their counterparts working day shifts (111). Other research has demonstrated lower tolerance of non-standard work hours among workers with mood disorders such as anxiety (112). The presence of chronic conditions is believed to deplete the body of critical physiological and psychic resources, resulting in greater risk of fatigue (85). Moreover, the relationship between work hours and health is likely circular, as long-term exposure to excessive work hours has been identified as a significant causal factor in the onset of chronic health problems (113).

###### 2.3.3.6.4 Sleep habits

The need for good sleep hygiene practices is heightened among individuals working extended hours or shift work. Insufficient or poor sleep, often linked to inadequate recovery, can serve as a common pathway from long work hours, shift work and work stress to various forms of illness (114). Non-work factors such as domestic and caregiving responsibilities can significantly impact the opportunity for recovery, as well as the quality and duration of sleep during recovery periods. Competing work and family responsibilities give rise to physical and psychological strain that can exacerbate fatigue (115). Studies investigating the effects of work scheduling on nurses have identified work-family conflict as a significant predictor of poor sleep quality (116). Additionally, sleep disruption resulting from the strain of work-life conflict has been demonstrated to have deleterious impacts on nurses’ mental health (115, 117).

###### 2.3.3.6.5 Work demands/stressors

Hectic work characterized by high work demands under fast-changing circumstances is physically and psychologically draining and can lead to high levels of fatigue in nurses (118, 119). Work that is hectic or physically strenuous is linked to poor sleep quality with deleterious effects on recovery (96). A synthesis of studies on work-related fatigue reported strong positive associations between work demands and the need for recovery across six different occupations (120). Nurses typically contend with heavy physical workloads and circumstances that demand a high level of emotional regulation to sustain attention and focus. The level of intensity of work demands in nursing can accelerate the onset of occupational fatigue and attendant fatigue-based risks (121). Nurses working in acute care environments, particularly those with a high proportion of patients at risk of rapid deterioration, often experience heightened strain and are more susceptible to fatigue (122, 123). Consequently, research on managing stress and fatigue in nurses recommends monitoring individual nurses’ level of fatigue during a work shift, as well as utilizing tools to predict fatigue risk (118, 124).

###### 2.3.3.6.6 Work schedule

Shift work, especially rotating schedules and night shifts, is known to contribute to poor sleep quality and work-related fatigue (42, 74). Long work shifts of 12 h or more have been linked with various fatigue-related safety risks (23, 25, 47), particularly shifts occurring at night (54). The number of consecutive work shifts and total weekly work hours are also associated with an increased risk of work-related accidents and injuries in a cumulative fashion (58, 125). One aspect of nurses’ work schedules that has been linked with higher risk of fatigue is the “quick return,” where nurses are given less than 11 h between the end of one shift and the start of the next. Considering optimal sleep periods (typically recommended as seven to eight hours for most adults), schedules that mandate quick returns to work do not allow for adequate recovery between shifts (118). Research has linked quick returns to a higher incidence of sick leave, a relationship likely influenced by the reduced amount of time nurses have available to sleep and recover from fatigue (126).

The significant positive effects of scheduled rest breaks during a shift are well-documented. In their study of workers hospitalized with severe injuries, Lombardi et al. (127) demonstrated that even minimal rest breaks of less than 30 min had a significant effect on delaying the onset of work-related injury (127). Likewise, Tucker et al. (128) reported that regular rest breaks constitute an effective method of controlling the accumulation of injury risk during a work shift (128).

## 3 Regulations on work hours and fatigue-risk management within safety-critical industries

The impact of long work hours on occupational health and safety outcomes is a major concern for employers, leading to the increased adoption of regulations across various industries (25). Governments in developed countries have increasingly set limits on work hours to minimize the adverse effects of fatigue on occupational health and safety outcomes. A review of these policies suggest that for the most part these conform to the research evidence on work hours safety; however, there are some notable exceptions, including within the Canadian health care sector.

A review of the regulations on work hours within Canada focused on the active regulatory limits enforced by government or another regulatory body within safety-sensitive industries including, transport, nuclear, aviation, health care. Policies concerning the safety limits on work hours within the US and the EU focus on restrictions enforced by regulatory bodies within the health care sector.

### 3.1 Work hours regulations in safety-sensitive industries in Canada

In Canada, certain classes of workers within federally regulated safety-sensitive industries are subject to specific restrictions on work hours. Motor vehicle operations/trucking, rail, nuclear and aviation are each governed by a set of restrictions on work hours to help maintain worker performance, forestall the onset of fatigue, and reduce the risk of accidents. The specific policies regarding work hours for each domain are described in turn. In general, work hours regulations focus on three key aspects of the work schedule: (1) total number of consecutive work hours; (2) number of successive [night] shifts; and, (3) mandatory minimum periods for recovery.

#### 3.1.1 Motor vehicle operators

Under Part III of the Canada Labour Code (CLC), motor vehicle operators are governed by the Motor Vehicle Operators Hours of Work Regulations (CRC, c.990). This classification encompasses city and highway vehicle operators involved in interprovincial and international transport of goods or passengers, as well as the transport of mail, and bus operators (129). This category includes commercial vehicles and trucking. The general limits on work hours for each class of worker as stipulated by the regulations are provided in Table 3.

**Table 3:** Motor vehicle operators hours of work regulations by class of worker.

Class of worker	Standard hours after which overtime is payable daily	Standard hours after which overtime is payable weekly	Maximum hours	Mandatory rest period
Highway Motor Vehicle Operators	n/s	60	13 h daily	10 h (8 h must be consecutive)
City Motor Vehicle Operators	9	45	13 h daily	10 h (8 h must be consecutive)
Bus Operators	8	40	13 h daily	10 h (8 h must be consecutive)

Notes: All regulations are based on safety scheduling for “Driving South of Latitude 60°N.” CRC denotes: Consolidated Regulations of Canada. Source: Canada Labour Code (129).

n/s, none specified.

#### 3.1.2 Railway operators

Transport Canada has set limits on work hours to protect the health and safety of railway operators under the Railway Safety Act. These types of employees include: locomotive engineers, conductors, trainmen, yardmen, pilots, operators of remote-control locomotives, and operators of light rail passenger equipment.

Notably, unlike regulations covering motor vehicle operators, legislation regarding work hour limits for railway operators specifically identifies its purpose as “fatigue management” so as to mitigate “fatigue-related risk that may affect safe railway operations.” Fatigue management is considered a “shared responsibility” between the railway company and the employees. In turn, the term “fit for duty” with respect to work hours limits and rest breaks appears multiple times in the legislation. An objective measure of fatigue/duty-fitness is taken prior to shift periods using the Karolinska Sleepiness Scale, a ten-item self-report measure of one's current level of fatigue (130). Specific directives of the regulation are given in Table 4.

**Table 4:** Transport Canada railway safety act, work hour provisions for employees.

Maximum duty period	Minimum rest period	Other provisions
- 12 h daily - 60 h weekly	- 12 h at home terminal - (10 h away from home terminal) - Within each rest period, 8 h must be undisturbed by the company	- Mandatory fatigue management plan (FMP) to be implemented by all railway companies

Source: Transport Canada (130).

Relevant excerpts from the policy as to specific strategies for fatigue management include:

Where an employee's duty period is scheduled to last more than 10 h and to end between 00:01 and 06:00, an employee shall report to the railway company, in accordance with the company's fatigue management plan, that they believe themselves to be fit for duty in accordance with the fatigue self-assessment training provided by the railway company and that they have met the following requirements:

• Obtained at least five hours of sleep in the 24 h prior to commencing the duty period;
• Obtained at least 12 h of sleep in the 48 h prior to commencing the duty period; and,
• Assessed themselves as scoring a seven or lower on the Karolinska Sleepiness Scale (scored on 1–10 scale, where is “extremely alert” and 10 is “extremely sleepy”).

In an effort to achieve maximal mitigation of fatigue-based risk, the policy also explicitly allows for “use of controlled napping protocols,” where appropriate (130).

#### 3.1.3 Nuclear

The work hours of employees in the nuclear energy industry are governed by the Canadian Nuclear Safety Commission under the Nuclear Safety and Control Act (NSCA). Like the railway industry, regulations for nuclear employees stipulate that the goal is to ensure “fitness for duty” by effectively “managing worker fatigue.” In particular, the regulation requires that nuclear operators “shall document and implement limits on hours of work and recovery periods that:

1. Provide sufficient time for sleep daily
2. Restrict consecutive shifts to limit the build-up of sleep debt
3. Provide sufficient time off to allow for recovery from sleep debt
4. Limit average weekly hours as a safeguard against cumulative fatigue.”

Moreover, nuclear operators must “document the rationale that justifies their limits on hours of work and recovery periods” and that “the rationale shall be based on scientific principles and knowledge.” In turn, it is required that all nuclear operators must “define and implement a range of measures to manage risks associated with fatigue, including those to manage the level of fatigue workers experience at work and to reduce the likelihood and consequences of fatigue-related errors” (131). The range of measures recommended for mitigating worker fatigue include: allowing rest periods or an opportunity to sleep, nurturing an environment that includes self-reporting when workers believe they are too fatigued to perform their duties competently and safely; employing additional supervisory oversight and independent verification when the risk of fatigue is highest (e.g., during the night shift, near the end of a shift, working beyond 12 h); rotating workers between tasks of varying cognitive and physical workloads; and, scheduling safety-critical tasks outside of peak times for fatigue (especially between the hours of 2:00 a.m. and 6:00 a.m.—referred to in the research as the “window of circadian low”). Specific limits on work hours as set forth by the NSCA are outlined in Table 5.

**Table 5:** Nuclear safety and control act, work hour provisions for employees.

Maximum duty Period	Minimum recovery period	Other provisions
- 16 h daily - 60 h weekly	- 72 h of rest must follow a block of four consecutive night shifts - 48 h of rest must follow two consecutive night shifts	- Maximum of 6 shifts on consecutive days - Maximum of 4 consecutive nights for 10 and 12 h shifts - Maximum of 54 h per week averaged over a fixed period of 13 weeks - Work shifts are not to exceed 12 h, especially night shifts

Source: Canadian Nuclear Safety Commission (131).

#### 3.1.4 Aviation

Work hour limits for air crews and air traffic controllers are governed by Transport Canada's Canadian Aviation Regulations (CAR). As part of a comprehensive approach to safety management, CAR also requires that all air operators have a fatigue risk management program in place for flight crew members as part of a comprehensive approach to safety management. Table 6 provides an overview of work hour restrictions and mandatory rest periods set forth in the regulation.

**Table 6:** Canadian aviation regulations, provisions for work hours by class of worker.

Category	Maximum duty period	Minimum recovery period	Other provisions
Pilots and aircrew	- Flight duty not to exceed period greater than 13 h in a 24 h period - For long range flights operated by un-augmented flight crews and where any part of the flight duty period infringes the WOCL, no flight may follow a scheduled flight of more than 7 h - Set maximum of 70 h in 7 consecutive days	- Must be provided with 120 consecutive hours free from duty, including five consecutive local nights’ rest in any 504 consecutive hours	- Minimum 15 min break every six hours of flight duty
Air traffic controllers	- Maximum of 10.5 h duty-time in a 24 h period	- Minimum of three consecutive days of rest between shifts	- 34/22 shift cycle comprised of 34 days of work and 22 days of rest (over a 56-day period); shift cycles consist of 5 days on, three days off

Source: Government of Canada (132).

The regulations also require that air operators implement a specific protocol for managing fatigue risk in employees—i.e., Fatigue Risk Management System for the Canadian Aviation Industry. The policy stipulates that, among other requirements, employers must provide aviation workers training in each of the following areas: personal fatigue management strategies relating to sleep hygiene, lifestyle, exercise and diet; sleep requirements and the science of fatigue; how to recognize fatigue in themselves and others; awareness of human and organizational factors causing fatigue such as sleep quality and duration, the impact of shift work and overtime, and the effects of changes in time zones.

#### 3.1.5 Health care

At present, there are no federal regulations governing the work hours of health care employees. The work hours of medical residents, however, are governed through negotiated agreements between the Provincial Residents’ Associations (PRAs) and employers. The PRAs currently set limits for residents at between 24 and 26 consecutive work hours. The exception is Quebec where, since July 1, 2012, residents’ time on duty has been restricted to 16 h due to result an arbitration ruling in the province that a 24 h duty period is a violation of the Canadian Charter of Rights and Freedoms and the Quebec Charter of Human Rights and Freedoms (133). Weekly work hour limits vary widely across provinces, ranging from 60 to 90 h per week (134). General regulations and restrictions pertaining to resident duty hours in Canada are outlined in Table 7.

**Table 7:** Canadian medical residents’ work hours regulations.

National regulations	Regulatory oversight	Duty hour restrictions
No	Work hours are currently regulated primarily through negotiated agreements between provincial residents’ associations (PRAs) and employers. In future, work hours may also be regulated via residency accreditation mechanisms.	- Variability in maximum hours worked per week (range 60–90 h) - Limit of 24–26 consecutive duty hours; exception is Quebec (2011) where limit is 16 consecutive work hours - limit of in-house call once every 4 days, averaged over four weeks - limit of out-of-house call once every 3 days

Source: Pattani et al. (134).

Like medical residents, currently there is no pan-Canadian limitation on nurses’ work hours. Where regulations exist, these are primarily through collective agreements with employers. There are currently dozens of collective agreements in effect within each province covering a range of nurse practice types—e.g., hospital, community health, and long-term care. Table 8 provides an overview of the key policies with respect to work hour limits contained within the master agreements for each province.

**Table 8:** Work hours provisions within nurses’ collective agreements by province.

Province	Maximum duty periods	Mandatory rest periods	Other provisions
British Columbia	- No maximum shift length - No more than 6 consecutive shifts (no more than 4 consecutive 12 h shifts)	- Two rest periods of 15 min during a standard shift, three rest periods for shifts of 10 or more hours - 30 min meal period for each 5 consecutive hours of work - At least one weekend off every 3 weeks	- Right to decline unreasonable overtime, except in emergency conditions
Alberta	- Maximum shift length of 16 h - No more than 6 consecutive shifts - No more than 2 consecutive weekends on duty	- Two 15 min rest periods and one 30 min meal break per 7.75 h shift; additional rest periods for extended shift lengths - At least 15 scheduled hours off duty between 7.75 h shifts - At least 2 consecutive days of rest - In the case of overtime and call back, 8 h off in the 12 h preceding the next shift	- Two consecutive days of rest is further defined between shift transitions (i.e., day shift to day shift is 63.75 h off duty, night to day is 71.75 h off duty) - Right to refuse unreasonable overtime, except in an emergency
Saskatchewan	- No maximum shift length - 15 h off duty between shifts (12 h for 12 h shifts) - No more than 6 consecutive shifts (no more than 4 consecutive 12 h shifts)	- Two 15 min rest periods for shifts lasting at least 6 h - 60 min rest during 12 h shifts - 2 consecutive days off per week - Minimum of 3 weekends off in a 6-week period	- Right to refuse overtime, except in emergency circumstances
Manitoba	- Maximum shift length of 16 h - Maximum of 7 consecutive shifts	- Minimum of 15 h between standard shifts - Minimum of 47 h off duty during a rotation period	
Ontario	- No maximum shift length - No more than 7 consecutive shifts for standard shifts (7.5 h) - No more than 3 consecutive shifts for extended tours (11.25 h) - No more than 4 consecutive shifts for innovative 2 days/2 nights (11.25 h) rotations - Maximum of 75 h bi-weekly	- 30 min unpaid meal break and two 15 min rest breaks during a standard shift - 45 min unpaid meal break and another 45 min rest break during extended tours - At least 48 h off when changing from nights to days - 15–16 h off duty following a standard shift (7.5 h) - 11–12 h off following an extended shift (11.25 h)	- Right to refuse overtime, except in an emergency - Some collective - Agreements allow for 8 to 12 h off with or without pay after a call back when on standby
Quebec	- Maximum shift length of 12 h - Maximum of 7 consecutive shifts	- Two 15 min rest periods per shift	
New Brunswick	- Maximum shift length of 16 h (unless due to unforeseen emergency) - Maximum of 7 consecutive shifts	- Minimum of 8 h between shifts	- In the event of working over 16 h due to unforeseen emergency, employer shall complete documentation before employee begins working - Overtime allowed though no employee shall be required to work a double shift
Nova Scotia	- Maximum shift length of 16 h - No more than 7 consecutive standard shifts; no more than 4 consecutive extended shifts - No more than 5 consecutive evening or night shifts - Maximum of 75 h bi-weekly	- Minimum rest period of at least 16 h between standard shifts (12 h between extended shifts) - Minimum of 2 days off each week - At least one weekend off every 3 weeks	- Rest interval after call back for nurses working on-call that provides for a rest interval of 8 h between the time the on-call shift is completed and the commencement of the nurse's next scheduled shift
Prince Edward Island	- No maximum shift length - No more than 7 consecutive shifts - No requirement to work double shifts without employee consent	- Two 15 min rest periods on each shift - At least 16 h between shifts (12 h for 12 h shifts) - At least 2 consecutive days off each week - Every second weekend off	- No requirement to work double shifts
Newfoundland and Labrador	- No maximum shift length - No more than 7 consecutive standard shifts; no more than 6 consecutive evening or night shifts - No more than 3 consecutive 12 h shifts - Maximum of 75 h every 2 weeks	- At least 16 h between shifts (12 h for 12 h shifts) - At least 2 consecutive days of rest per week - Two weekends off per month	- No requirement for double shifts

Source: Canadian Federation of Nurses Unions (CFNU) (135).

Notably, although five provinces—Alberta, Manitoba, Quebec, Nova Scotia, and New Brunswick—only Quebec sets the limit at 12 h, which the literature suggests is the safety maximum. Still, Quebec allows for up to a maximum seven consecutive work shifts, which the evidence indicates could be problematic for nurses working extended shifts. Only Nova Scotia and Newfoundland and Labrador have limited the number of consecutive night or evening shifts. Four provinces—British Columbia, Alberta, Saskatchewan and Ontario—have explicitly provided for the right to refuse overtime hours, though New Brunswick, PEI and Newfoundland and Labrador have agreements that stipulate no requirement to work double shifts. Four provinces, Ontario, Nova Scotia, PEI and Newfoundland have mandated a minimum break of 11 h (i.e., no “quick returns”) after an extended shift.

### 3.2 Work hours regulations in health care in the United States

Like its peer countries, in the United States the work hours of truck drivers, locomotive engineers, and pilots are regulated to protect the public from fatigue-related errors, though hospitalized patients lack similar protection (70). At present, there are no restrictions on the number of hours a nurse may voluntarily work in a 24 h or a seven-day period in the United States, while only minimal restrictions exist on hours worked by physicians (70).

As of 2011, the Accreditation Council for Graduate Medical Education (ACGME) recommended the following work hour limits for medical residents: interns were allowed a maximum of 16 continuous work hours, with an overall workweek limit of 80 h; shifts could last up to 24 h, plus an additional six hours for transitioning care; residents were entitled to one day off per week, averaged over a four-week period; and on-call duties could be scheduled no more than once every three nights (136).

In contrast, nurses’ work hours are regulated by state governmental agencies known as nursing regulatory bodies (NRBs), leading to variations in work hour limits across states. It should be noted, however, that none of these jurisdictional restrictions address the duration nurses may work on a voluntary basis (70). An overview of work hours regulations for health care professionals in the US is provided in Table 9.

**Table 9:** Work hours regulations for health care professionals, select jurisdictions (United States).

Class of worker	Regulatory body and type of regulation	Maximum work hours in 24 h period and/or 7-day period	Minimum rest period	Other provisions
Medical Residents	- No federal regulations	- May not work more than 80 h per week or 24 consecutive hours	- Must have at least 10 nonworking hours between shifts	- No prohibitions on working extra hours (moonlighting)
Registered Nurses	- No federal regulations	- May not work more than 16 h in any 24 h period (West Virginia) - No more than 14 consecutive hours on duty (Alaska) - No more than 12 consecutive hours on duty (California, Maine, Massachusetts, Minnesota, New Hampshire, Oregon, Rhode Island) - Limit of 48 h per week (Oregon)	- If a nurse works after 8 consecutive hours, must be given at least 10 h off before the next shift (Maine) - If a nurse works after 12 consecutive hours, must be given at least 8 h off before the next shift (Illinois, New Hampshire)	- Prohibited mandatory overtime (Alaska, California, Illinois, Maine, Maryland, Massachusetts, Minnesota, New Hampshire, New Jersey, New York, Oregon, Pennsylvania, Rhone Island, Texas, Washington, West Virginia)

Sources: Burchiel et al. (70), National Council of State Boards of Nursing (NC SBN), (136), Page (137).

Research on the impact of state regulations on nurses’ work hours demonstrates their effectiveness in reducing overwork (138, 139). In one study, Bae and Yoon (139) found that government regulations on mandatory overtime and consecutive work hours led to a 3.9 percentage point decrease in the likelihood of mandated overtime and an 11.5 percentage point reduction in the likelihood of nurses working 40 h per week (139).

### 3.3 Work hours regulations in health care in Europe

As in all safety-sensitive industries in the EU, work hours in health care are regulated by the European Working Time Directive (EWTD). Instituted in 1993, the EWTD introduced specific measures for scheduling shifts and rest periods to limit work hours, based on evidence that shift work and excessive hours can pose significant occupational health and safety risks (95). The work hours of both medical residents and nurses are governed by the EWTD. The specific requirements of this legislation are detailed in Table 10.

**Table 10:** Work scheduling requirements of the European working time directive (EWTD).

Maximum duty hours	Mandatory rest period
- Maximum of 8 h of work if categorized as a night worker - No more than 48 h per week, including overtime	- 20 min of continuous rest every 6 h - 11 h continuous rest in every 24 h period - Every 7 days a minimum of 24 uninterrupted hours in addition to 11 consecutive hours of rest

Source: Government of the United Kingdom (140).

## 4 Discussion

A rapid review of the evidence regarding the occupational health and safety impacts of excessive work hours is both clear and compelling. Extended shifts exceeding 12 h significantly increase the risk of occupational fatigue, leading to various fatigue-related hazards. Likewise, working more than 40 h per week is associated with higher health and safety risks. The cumulative effect of work-related fatigue raises the likelihood of accidents and injuries, both during work and after shifts (e.g., due to drowsy driving). Chronic fatigue also negatively impacts workplace culture and team cohesion, as fatigued employees often show reduced empathy toward peers and a lower willingness to cooperate. The health consequences of excessive work hours include sleep disturbances, mood disorders, and serious chronic conditions such as cardiovascular disease, metabolic disorders, and cancer. Fatigue from extended work hours is further exacerbated by shift work, especially rotating schedules that involve night shifts, which disrupt circadian rhythms. The body's neurophysiological rhythms, particularly during the “window of circadian low” (2:00 a.m.–6:00 a.m.), reduce the ability to work effectively at night, leading to a higher risk of workplace injuries and long-term health problems for night shift workers. Extensive research, particularly in nursing, has highlighted the detrimental effects of long work hours and shift work schedules on both personal and patient safety.

Despite the well-documented occupational health and safety risks linked to long work hours and occupational fatigue—risks that have prompted governmental regulations in safety-critical industries like transportation, aviation, and the nuclear sector—the health care industry remains largely unregulated in this area. In Canada, the only exception is the work hour restrictions for medical residents, enforced by provincial regulatory bodies. However, there are no governmental restrictions currently exist on the number of hours a nurse can work within a 24 h period or over a 7-day interval. Protections against overwork for nurses are provided solely through individual collective agreements, within only five provinces placing limits on shift-length under normal (non-emergency) conditions. Of these, only Quebec enforces a 12 h maximum, which aligns with evidence-based recommendations. Considering the longstanding history of regulatory limits on work hours in safety-critical industries outside of health care, the lack of governmental protections for nurses’ working conditions raises the question of gender-bias as a potential underlying factor. It is crucial to bring this disparity to the forefront for policy makers, not only in the interest of equity, but also to ensure public safety. In Europe, comprehensive worker protections regarding work hour limits set a valuable precedent for other Western countries, and could offer a straightforward solution in this context.

Additionally, fatigue risk management programs are standard practice within safety-critical industries and should be adopted in health care settings as well. Occupational health and safety management systems such as the ISO-45001 provide specific guidelines for employers to identify potential fatigue hazards and strategies to mitigate fatigue risks. These standards encompass crucial safeguards related to work hour limits, including shift duration, properly timed rest breaks, the number of consecutive shifts, weekly work hour loads, and systems for fatigue management and monitoring. Fatigue monitoring enables employers to consider individual factors like age and health status, which can vary over time. These frameworks also provide guidance on educating workers to understand the causes and consequences of fatigue, as well as how to recognize fatigue in themselves and others. Health care employers can reinforce these standards by adopting practices such as providing designated napping spaces for nurses working extended or overnight shifts and offering transportation home for those fatigued after their shifts. These actions help reduce the risks associated with fatigue, enhance overall safety, and improve working conditions for nurses—key steps in addressing staffing shortages by boosting both retention and recruitment.

This review marks the first direct application of research evidence on the occupational health and safety impacts of excessive work hours and occupational fatigue to gain a clearer understanding of safe work hour limits in nursing. It also offers a foundational framework for designing work schedules and establishing work hour targets to promote safe nursing practices. The primary strength of the review lies in its comprehensive approach, synthesizing findings from the academic literature and linking them to current policy recommendations across multiple safety-critical sectors beyond health care. Further research on the effects of long work hours in nursing could, in the future, enable a more extensive systematic review of the evidence. However, a limitation of this review is the short timeframe allotted for the study—less than six months—arising from the urgent need to deliver a rapid overview of the evidence to Canadian health care regulators. Additionally, the involvement of only a single investigator could be considered a weakness, as the absence of multiple reviewers may have reduce the capacity to check for potential bias. In the future, additional studies on the occupational health and safety consequences of long work hours should also include qualitative investigations into workers’ experiences with work hour regulations, particularly assessing the extent to which these regulations are enforced in practice. Furthermore, research on resilience among nurses (141) should place greater emphasis on working conditions and work hours as key factors contributing to psychological harm and reduced well-being.

Still, given the well-documented effects of excessive work hours on both individual and public health, there is ample evidence supporting the need for stricter regulations on nurses’ work hours. Ensuring safe and high-quality care over the long term requires providing nurses with robust support systems and implementing fair scheduling practices. These measures will not only improve nurses’ work satisfaction and overall wellbeing but also help address staffing shortages by fostering better working conditions. This, in turn, creates a virtuous cycle that leads to better patient outcomes and a more resilient health care system.
